# Rayleigh–Taylor instability in strongly coupled plasma

**DOI:** 10.1038/s41598-022-15725-2

**Published:** 2022-07-07

**Authors:** Rauoof Wani, Ajaz Mir, Farida Batool, Sanat Tiwari

**Affiliations:** Department of Physics, Indian Institute of Technology Jammu, Jammu, 181221 India

**Keywords:** Plasma physics, Astrophysical plasmas, Laser-produced plasmas

## Abstract

Rayleigh–Taylor instability (RTI) is the prominent energy mixing mechanism when heavy fluid lies on top of light fluid under the gravity. In this work, the RTI is studied in strongly coupled plasmas using two-dimensional molecular dynamics simulations. The motivation is to understand the evolution of the instability with the increasing correlation (Coulomb coupling) that happens when the average Coulombic potential energy becomes comparable to the average thermal energy. We report the suppression of the RTI due to a decrease in growth rate with increasing coupling strength. The caging effect is expected a physical mechanism for the growth suppression observed in both the exponential and the quadratic growth regimes. We also report that the increase in shielding due to background charges increases the growth rate of the instability. Moreover, the increase in the Atwood number, an entity to quantify the density gradient, shows the enhancement of the growth of the instability. The dispersion relation obtained from the molecular dynamics simulation of strongly coupled plasma shows a slight growth enhancement compared to the hydrodynamic viscous fluid. The RTI and its eventual impact on turbulent mixing can be significant in energy dumping mechanisms in inertial confinement fusion where, during the compressed phases, the coupling strength approaches unity.

Rayleigh–Taylor instability (RTI)^1,2^ occurs in a fluid system in which a heavier fluid (density, $\rho _h$) lies on top of a lighter fluid (density, $\rho _l$) under the effect of the gravity^3,4^. As it evolves, the modes at the fluid interface grow in amplitude, forming bubbles that rise due to buoyancy and spikes, which fall due to the gravity, eventually leading to turbulent mixing^5^. The instability is a primary mixing mechanism in supernovae explosions^6,7^, solar corona^8^, volcanic eruptions^9^, tokamaks^10^, Bose–Einstein condensate (BEC)^11,12^, paramagnetic fluids^13,14^, laser generated high-energy-density (HED) plasmas^15,16^, and inertial confinement fusion (ICF)^17,18^ covering multiple orders of length scales. Usually, hydrodynamic models explain the RTI for fluids, whether neutral or charged, using the Navier–Stokes (NS) model without or with Maxwell’s set of equations. This paper focuses on RTI growth and its nonlinear evolution in strongly coupled plasmas (SCP). Under strong inter-particle correlations, these plasmas reflect visco-elastic nature that can not appropriately be represented using the standard hydrodynamic model. Also, kinetic effects become significant enough to influence the continuum effects in such scenarios. We employ a classical two-dimensional (2D) molecular dynamics (MD) model to study the growth and mixing properties of RTI. The work highlights the impact of strong inter-particle correlations and includes contributions from all scales, including thermal fluctuations.

In the recent past, MD simulations have been carried out at a microscopic level to study several hydrodynamic instabilities such as Kelvin–Helmholtz instability (KHI)^19,20^, RTI^21–23^, Rayleigh–Bénard instability^24^, and bump-on-tail (BOT) instability^25^. Kadau et al.^21^ first carried out a three-dimensional (3D) MD simulation for RTI in Lennard–Jones (LJ) fluids. Their results, in general, matched with linear stability analysis of the Navier–Stokes model and paved the way to explore mixing at microscopic scales. Further, Ding et al.^23^ carried out RTI studies for Ar/He interfaces through LJ pairwise interactions. The work suggested the considerable difference in the formation and evolution of spikes at the microscopic level to the macroscopic scale. It also showed the detached droplet formation due to the thermal fluctuations. In both the works mentioned above, the focus was primarily on the role of microscopic fluctuations. Our focus is towards systems comprising a large number of charged particles, where dynamics is governed by the Coulomb force. As surrounding charges shield each charged particle, the effective pairwise potential takes the form of Yukawa/Debye–Hückel interaction potential given by^26^

1 $$\begin{aligned} \phi _{ij} =\ \frac{1}{4\pi \epsilon _0}\frac{q^2}{r_{ij}}\exp {(-r_{ij}/\lambda _D)}. \end{aligned}$$

Here $r_{ij}$ is the distance between the *i*th and *j*th particles, *q* is the charge on each particle and $\lambda _D$ is the Debye screening length. The Yukawa/Debye–Hückel fluids in nature include soft-matter systems e.g., charged colloids^27,28^ and concentrated protein systems^29^, strongly coupled plasmas e.g., quark-gluon plasma^30,31^, and dusty plasma^32^, and many ionic-liquids^33,34^. Using the potential of the form Eq. (1), we have modelled RTI in SCPs. With known appropriate pairwise interaction, the MD provides the most fundamental and comprehensive picture of a system’s micro-and macroscopic dynamical process. The advantage of MD is that it is based on the fundamental nature of the forces. Physical processes such as shear thinning^26^ and negative entropy production^35^ having their origin at fluctuations in natural fluids are missed in the most hydrodynamic models. The micro-scale fluctuations captured by MD allow us to probe the emergence of macroscopic hydrodynamic quantities as the averages of these micro-scale fluctuations inherently. Moreover, the purpose of carrying out MD is to overcome one of the limitations of hydrodynamic models in incorporating strong inter-particle correlation effects. Correlations let the viscous liquids reflect solid-like properties usually characterised as a family of visco-elastic fluids. Electrolytes, ionic liquids, and plasmas are the charged liquids that belong to this family, where individual particles interact via Yukawa/Debye–Hückel interaction potential. Especially plasmas, in extreme conditions (high charge on particles, extremely low temperatures, or at high density), reflect solid-like properties and also show the presence of transverse shear waves. Their solid-like reflection can be quantified through the coupling parameter, $\Gamma$ as:

2 $$\begin{aligned} \Gamma =\ \frac{\left\langle E_p \right\rangle }{\left\langle E_k \right\rangle }=\ \frac{1}{4\pi \epsilon _0}\frac{q^2}{ak_BT}. \end{aligned}$$

The coupling strength is defined as the ratio of average Coulomb potential energy $\left\langle E_p \right\rangle$ and average thermal kinetic energy $\left\langle E_k \right\rangle$. Here $a = (\pi n)^{-1/2}$ is the average inter-particle separation or Wigner-Seitz radius, and n is the areal number density. *T* is the temperature of particles. The Yukawa nature is quantified by screening parameter $\kappa = a/\lambda _D$. The limit $\kappa \rightarrow 0$ represents a pure Coulomb system, while the limit $\kappa \rightarrow \infty$ represents the hard-sphere like interactions. Two dimensionless parameters characterize the thermodynamic and transport properties of Yukawa one-component plasmas; the Coulomb coupling strength $\Gamma$ and inverse Debye screening length $\kappa$^36,37^. We found the enhanced correlations (i.e., the increase in $\Gamma$) suppress the RTI in Yukawa fluids. This result is supported by the findings of Das et al.^38^ and Avinash et al.^39^ that propose the reduced growth of instability with increasing coupling strength using a phenomenological generalized hydrodynamic (GHD) visco-elastic model. Their results from the GHD model suggest a decrease in growth rate as $\gamma = \sqrt{gkA_t - \eta /\tau _m k^2}$. Here, $\eta$ and $\tau _m$ are the viscosity and relaxation time, respectively, and both depend on the coupling strength $\Gamma$. In weakly coupled plasma, the growth rate attains the standard incompressible hydrodynamic limit $\sqrt{g k A_t}$^38,39^. Also, *g* is the acceleration due to the gravity, $A_t = (\rho _h - \rho _l)/(\rho _h + \rho _l)$ is the Atwood number to quantify the density gradient and *k* is the wave vector of the excited mode. The penetration depths of spikes $H_S(t)$ into the light fluid and bubbles $H_B(t)$ into the heavy fluid are usually governed quadratically in time $H_{S, B} =\gamma _{q_S, q_B} A_t g t^2$ using the inherent inviscid NS model. The quadratic growth rate of spike $\gamma _{q_S}$ and bubble $\gamma _{q_B}$ is found to be dependent and independent of the variation of the Atwood number $A_t$ respectively using the continuum NS model as well as the LJ atomistic simulations^40^. It is noteworthy that the viscosity for such strongly correlated mediums acts to support elasticity rather than playing a viscous damping role. We have also studied the effect of potential shielding over the instability explicitly through parameter $\kappa$. The shielding was found to increase the diffusive nature of the medium and the growth rate of RTI. Both these observations may be seen as a development of RTI on an effectively lower value of coupling strength which may be approximated as $\Gamma ^*= \Gamma \exp {(-\kappa )}$^41^.

While results in this paper are generalized and represent any fluid with pairwise Yukawa interactions, our terminologies and approach are inclined towards strongly coupled plasmas^42,43^. The SCPs include dusty plasma^44^, ultracold plasmas^45–48^ and dense plasmas^49^ depending on charge, temperature and density as factors responsible for strong correlations. In all three forms of SCPs, the interaction in bulk plasma is represented by shielded Coulomb potential. Our results could interest the inertial confinement fusion community as RTI is an unavoidable mixing mechanism in the ICF process. During the ICF process, the plasma has been claimed to be reaching close to moderate coupling strengths^50,51^. In such coupling regimes, the RTI growth should be lower than that predicted by hydrodynamics models.

## Results

### RTI: natural growth through maximally growing mode

**Figure 1:**
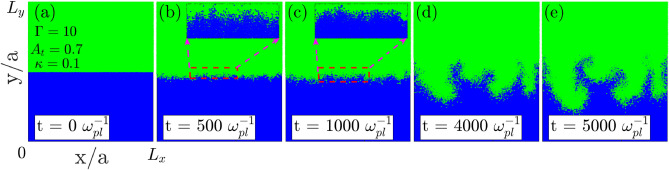
Time evolution of the RTI in a naturally evolved square system ($L_x = L_y$) through maximally growing modes. (**a**) The step density profile of the system, at time $t = 0\ \omega _{pl}^{-1}$, is unstable towards the RTI under gravity. (**b**–**e**) reflect growing modes at interface at times $t = 500$, 1000, 4000 and $t = 5000$ $\omega _{pl}^{-1}$, respectively. Inset in subplots (**b**,**c**) are magnified views showing early growth of modes in thermal fluctuations at the interface.

We let the equilibrated system evolve naturally after removing the partition between heavy (top) and light (bottom) fluids as in Fig. 1a. Initially, the instability grows from thermal fluctuations at the interface. The insets in Fig. 1b,c show the early stage growth of modes growing from fluctuations. Quickly, the maximally growing mode dominates due to the higher growth rate and becomes visible. At later stages of the evolution, the maximally growing mode (of the chosen system) typically corresponding to $k \approx 4k_0$ is visible in subplots Fig. 1d,e at times $t = 4000$ and $t = 5000$ $\omega _{pl}^{-1}$ respectively. Here $k_0 = (2\pi )/L_x$ is the fundamental mode at the interface in x-dimension. We observe the growth of mushroom clouds over the interface, a characteristic feature of RTI. The bubbles of lighter fluid (in blue) can be seen moving upwards against the gravity, and the spike of the heavier fluid (in green) penetrates, the lighter fluid downwards along the direction of gravity. A slight compression of the light fluid layer due to the early-stage free fall-like motion of the heavy fluid is visible during evolution from 0 to 500 $\omega _{pl}^{-1}$. But the same has no significant impact on characteristic RTI features. We have brought this effect to a negligible level in the rest of the simulations by adjusting the system dimension as $L_y = 10L_x$. We will remain confined to the same system configuration throughout the manuscript.

As the focus of present studies is to compare the growth rate dependence on various physical parameters of the strongly coupled plasma, RTI growing via natural modes is not suitable. Individual modes cannot be tackled precisely as they grow simultaneously in a naturally evolving system. As the prime motive is to observe the effect of $\Gamma$, $\kappa$ and $A_t$ on the growth, it is useful to fix the initial perturbation on individual mode dominantly. It also helps in capturing the early exponential dynamical regime. The early exponential growth (in linear regime) is hard to analyse. Moreover, it takes longer to explicitly see the maximally growing mode if the system evolves naturally. Thus, we will artificially perturb the system at the interface for the rest of the paper.

### RTI: growth through single-mode perturbation

**Figure 2:**
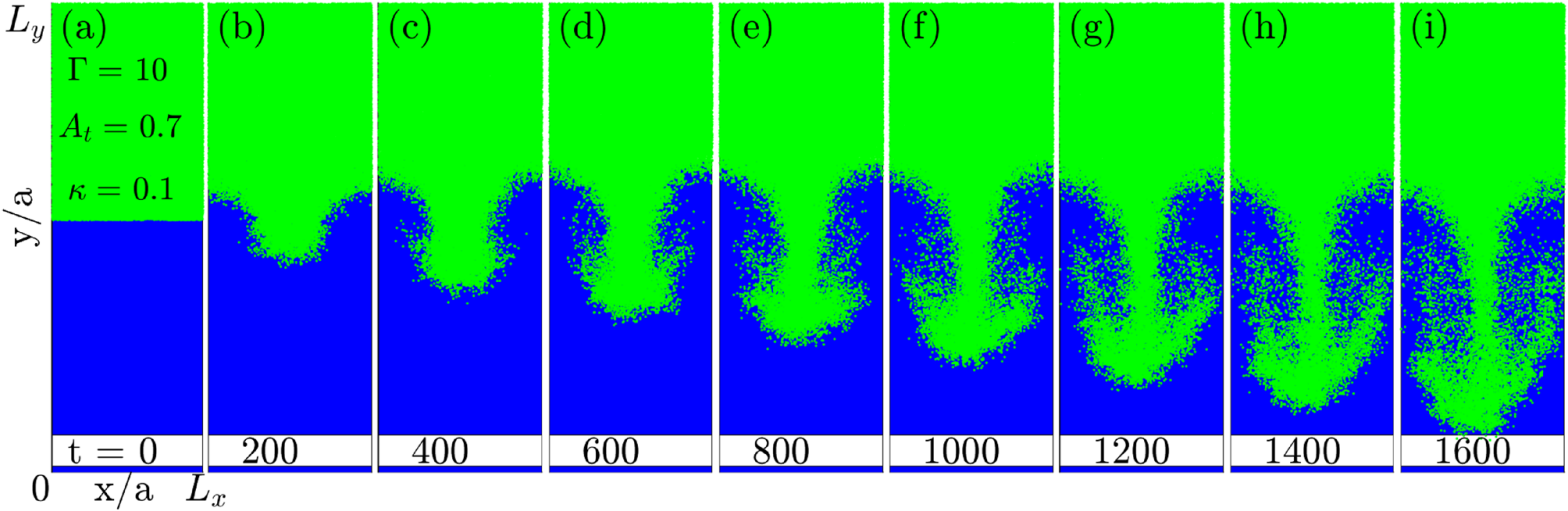
Time evolution of single-mode RTI. (**a**) The initial step density profile with $L_y = 10 L_x$. (**b**–**i**) are snapshots of growth of RTI at time intervals of 200 $\omega _{pl}^{-1}$ each. The rise of bubbles (blue-coloured) of light fluid due to buoyancy and fall of the spike (green-coloured) of heavy fluid due to gravity is observed. From (**e**) onward, the nonlinear mushroom cloud formation due to secondary KHI is visible.

To observe single-mode evolution, we perturb the interface with the mode $k_0 = (2\pi )/L_x$ that fit the system along x-direction. The sinusoidal perturbation added to the velocity of particles in a region of a few average inter-particle thicknesses at the interface as shown in Fig. 8b (“Methods”). The form of the velocity perturbation is $v_y = v_y^{thermal} + \xi _0 \cos {(k_x x)}$ with $\xi _0 = 1.5v_y^{thermal}$ and $k_x = k_0$. It takes about $10\ \omega _{pl}^{-1}$ for velocity perturbation to reflect in particle positions and hence in the density profile. Figure 2 shows the excitation of single-mode through perturbation and its evolution due to the RTI. Subplot Fig. 2b clearly shows a growing sinusoidal perturbation with wavelength corresponding to system width $L_x$. In time, as shown in subplots Fig. 2b–i, the sinusoidal perturbation grows with heavy fluid penetrating within the light fluid as a spike that eventually forms the mushroom structure at later stages. Simultaneously, the bubble of the light fluid grows upward into the heavy fluid. Though, the growth of the bubble is usually slow compared to the spike growth and attributed to the $A_t$. For larger values of $A_t$, the free-fall of the spike is expected, leading to higher growth of spike compared to the bubble.

**Figure 3:**
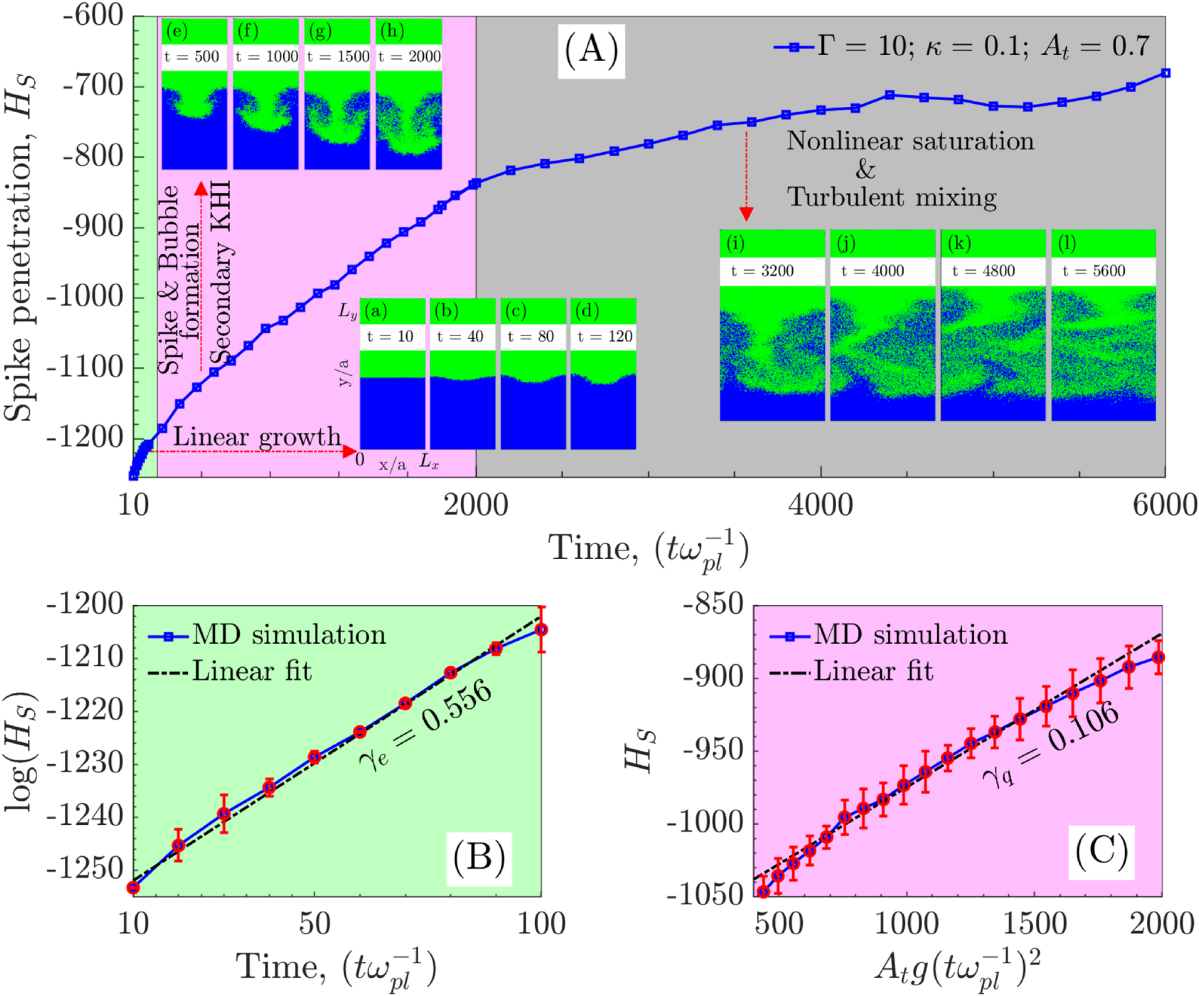
(**A**) Spike amplitude in single-mode RTI covering exponential and quadratic growth regimes and the nonlinear saturation stage. In subplot (**A**), the insets (**a**–**d**) show linear growth of modes, insets (**e**–**h**) shows the formation of spikes of heavy fluid and bubbles of light fluid and insets (**i**–**l**) show the nonlinear saturation that finally leads towards the turbulent mixing. (**B**) The exponential growth regime at the early-evolution stage, and (**C**) the quadratic growth regime during later evolution. The error bars in subplots (**B**,**C**) reflect the statistical error associated with the mean spike amplitude. An error has been calculated as the statistical variation in spike amplitude from its mean value for multiple simulation replicas for the same ensemble.

**Figure 4:**
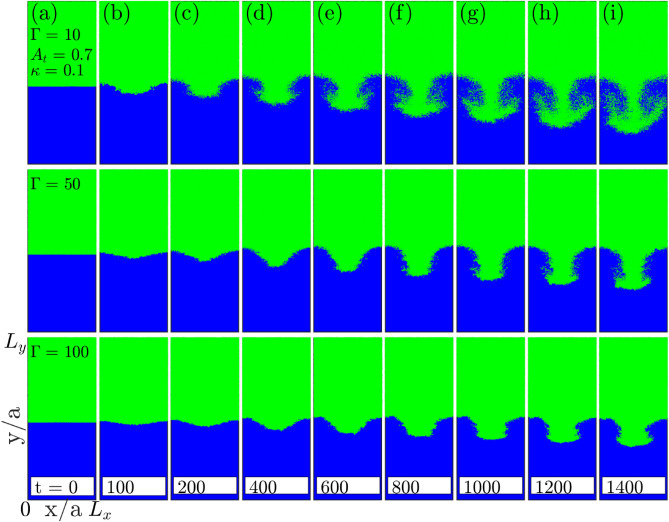
Effect of coupling strength $\Gamma$ on single-mode RTI. The top to bottom rows shows the evolution of single-mode RTI in systems with increasing coupling (Coulomb) strength. The suppression of growth of the spike amplitude in single-mode RTI is clearly visible with increase in the coupling strength. (**a**) In each row at $t=0\ \omega _{pl}^{-1}$, shows the initial step density profile. The snapshots in each row are shown at $t=100\ \omega _{pl}^{-1}$ (**b**) and rest all (**c**–**i**) with an interval of $t= 200\ \omega _{pl}^{-1}$, from $t= 200\ \omega _{pl}^{-1}$ onward upto $t= 1400\ \omega _{pl}^{-1}$s. The related movie is provided in Supplementary Material.

Figure 3A shows the spike penetration ($H_s = H_s (0) -H_s (t)$) within the light fluid for the system at coupling strength $\Gamma = 10$. Here $H_s(0)$ is the position of unperturbed interface i.e., $L_y/2$ and $H_s(t)$ is the position of the tip of spike in time. We have recorded the spike amplitude evolution for 6000 $\omega _{pl}^{-1}$s as shown in blue line with square marker. It broadly passes through the three dynamical stages, (1) exponential growth (green-colored), (2) spike and bubble formation with secondary KHI (red-colored) and (3) the nonlinear saturation leading to the turbulent mixing (black-colored) of RTI. In the first region (inset plots from (a) to (d)), the amplitude of the spike grows exponentially as per the linear stability analysis. An incompressible, inviscid hydrodynamic model^3^ suggests the RTI growth rate as $\gamma _e = \sqrt{g A_t k_x}$. Though in the present scenario, the viscosity and solid-like properties also play a significant role in deciding the growth rate. In the second region (inset plots (e) to (h)), the sinusoidal perturbation evolves nonlinearly into bubbles and spikes of lighter and heavier fluids, respectively. The secondary KHI that develops due to the shear velocity between two penetrating fluids gives rise to the formation of mushroom clouds. Finally, in the third region (inset plots (i) to (l)), the nonlinear saturation of the instability is observed as the spike amplitude almost stops growing. During this stage, turbulent mixing occurs, distributing the energy associated with excited/perturbed mode to smaller scales up to kinetic levels.

**Figure 5:**
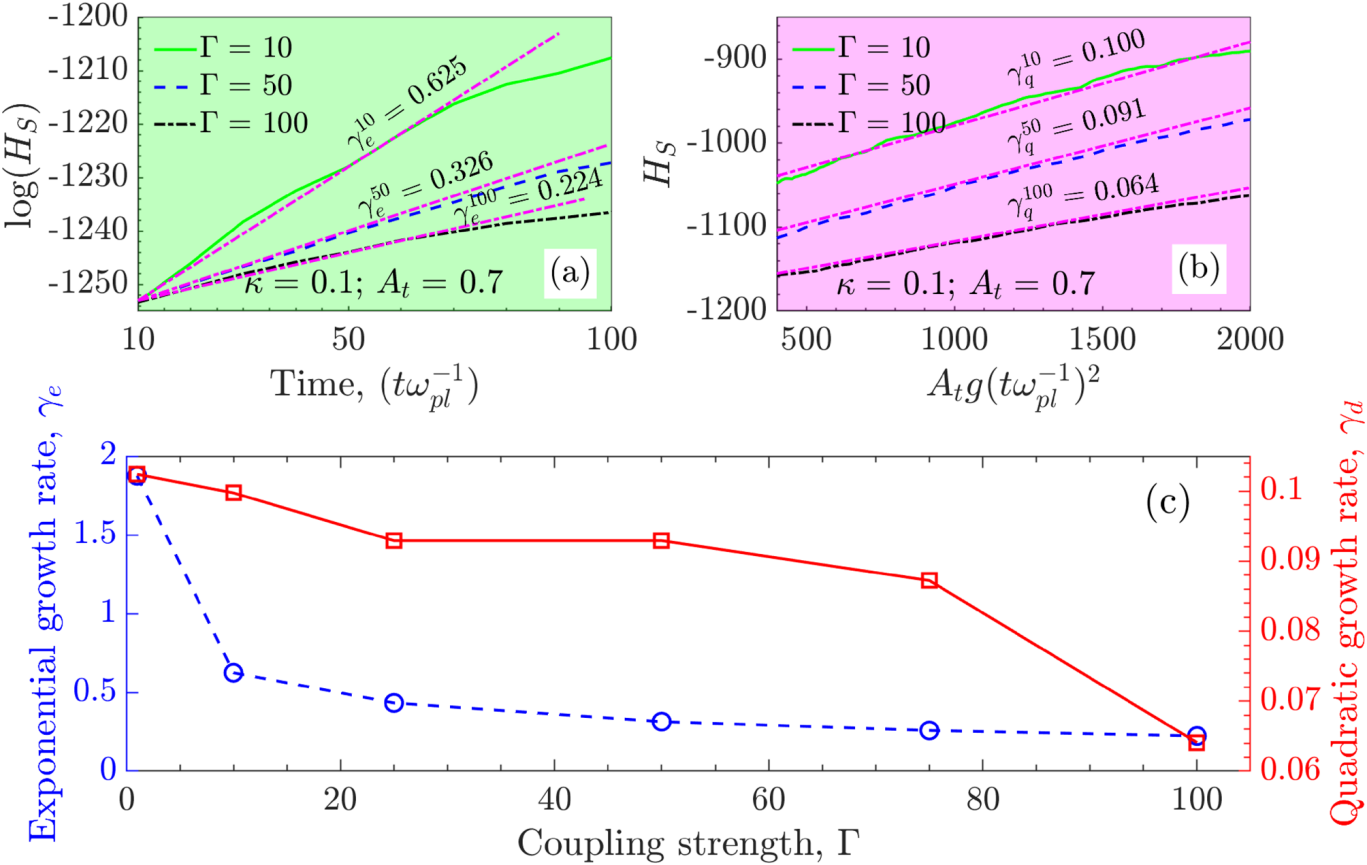
Effect of coupling strength $\Gamma$ on the growth rate of single-mode RTI. (**a**) Growth rate in exponential regime $\gamma _e$ and (**b**) growth rate in quadratic regime $\gamma _q$. Both show the same trend in the growth rate i.e., the decrease in growth with an increase in Coulomb coupling strength. (**c**) The exponential $\gamma _e$ and quadratic $\gamma _q$ growth rate dependence on coupling strength $\Gamma$.

We understand that being a kinetic simulation, there will be statistical fluctuations in the growth of RTI for different replicas of the same ensemble. It is computationally expensive to carry out multiple simulations with different initial particle configurations. Though, to provide an idea of the possible statistical error, we have attempted multiple simulations for a single case as in Fig. 3B,C. Each simulation starts with a different arrangement of position and velocity but the same $\Gamma = 10$, $\kappa =0.1$ and $A_t=0.7$. They all follow the same trend in growth, suggesting statistical error to be very small and have the least impact on RTI growth. This suggests that the statistically different replicas of systems do not impact the growth rate of the RTI. We have also plotted the error bar over the average growth rate obtained in each replica and finally best fitted to get the growth rate in each regime. Further, kinetic simulations make it challenging to demarcate a clear separation of linear growth, quadratic growth, and full nonlinear evolution. Though our best fits are a good representation in giving the message that the growth rate decreases with the increase in coupling strength $\Gamma$ without any ambiguity, as shown in Fig. 4.

#### Determination of growth rate in exponential regime $\gamma _e$ and quadratic regime $\gamma _q$ for single-mode RTI

The growth rate of instability is calculated by following the change in the fall (rise) of the spike (bubble) from the interface position. The growth rates of spike and bubble are typically different depending on the choice of the $A_t$. Our focus is limited to the growth rate of spikes only. The growth in spike amplitude is categorized in two regimes, namely (1) the exponential growth and (2) the quadratic growth^21^. The exponential growth ($H_S(t) = H_S(0) e^{\gamma _e t}$) observed during very early-time evolution of the unstable mode within first 100 $\omega _{pl}^{-1}$s. As the perturbation grows from fluctuations, $H_S(0)$ is the amplitude of spike at 10 $\omega _{pl}^{-1}$s when the single-mode starts visualizing. Figure 3B shows a linear slope as the growth rate of logarithmic spike amplitude changes. Further, during the late-time dynamics typically from 400–2000 $\omega _{pl}^{-1}$s, we observed the spike amplitude growth following the quadratic dependence over the time $H_S (t) = \gamma _q A_t g t^2$ in Fig. 3C. The exponential regime has a higher growth rate due to the abundance of free energy available at the early evolution stage in the system. During the quadratic evolution stage, the growth rate decreases because the instability is heading towards the nonlinear saturation stage due to the exhaustion of free energy available in the system. Though we have not explicitly calculated the free energy changes in the simulation, we understand that due to complex nature of nonlinearity not all the available free energy might get exhausted at the saturation stage.

### Effect of Coulomb coupling strength $(\Gamma )$ on the growth of single-mode RTI

Here, we estimate the growth rate of RTI at three different values of coupling strengths $\Gamma = 10$, 50 and $\Gamma =100$. Figure 4 shows the time evolution of instability in single-mode from subplots (a) to (i). Each row represents a different value of $\Gamma$ increasing from top to bottom. The snapshots of spike amplitude at different times clearly show the decrease in the growth rate of RTI as $\Gamma$ increases from 10 to 100. The exponential $\gamma _e$ and quadratic $\gamma _q$ growth rates for different coupling strengths have been calculated. We found the exponential growth rate $\gamma _e$ and the quadratic growth rate $\gamma _q$ both decreasing with the increase in coupling strength. Figure 5a shows the exponential growth rate for single-mode RTI at the early evolution stage that indicates a reduction in the growth rate with increasing $\Gamma$. Figure 5b shows the quadratic growth of single-mode RTI at a later evolution stage that also indicates a decrease in growth with an increase in $\Gamma$. The solid traits get prominent with increasing coupling strength $\Gamma$. Thus, the RTI growth rate reduces as the medium attains more and more solid-like properties. Figure 5c shows the RTI growth rate variations with coupling strength for single-mode perturbation. The exponential growth rate reduction with $\Gamma$ is significant compared to the quadratic growth rate.

**Figure 6:**
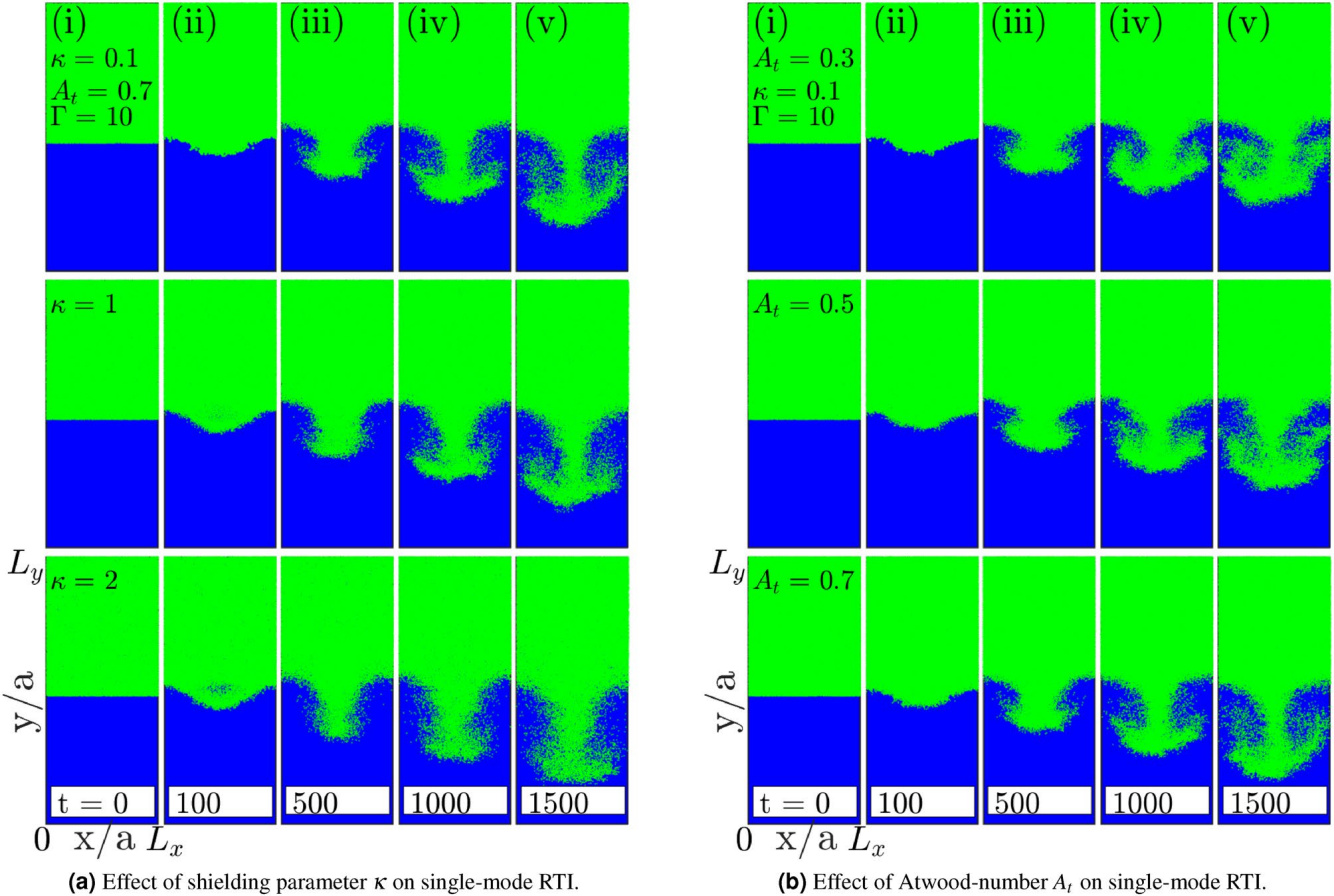
Effect of screening parameter $\kappa$ [sub-figure (**a**)] and Atwood number $A_t$ [sub-figure (**b**)] on single-mode RTI. In both sub-figures, subplots (**i**)–(**v**) show the time evolution of instability from linear ($t=100 \omega _{pl}^{-1}$s) to nonlinear stages $t=1500\ \omega _{pl}^{-1}$s. Sub-figure (**a**): first to third rows represent growth of RTI for $\kappa = 0.1, 1$ and $\kappa = 2$. Sub-figure (**b**): first to third rows represent growth of RTI for $A_t = 0.3, 0.5$ and $A_t = 0.7$.

### Effect of screening parameter $(\kappa )$ on the growth of single-mode RTI

In plasmas and electrolytes, the Coulombic interaction between charges is shielded by surrounding charged particles leading to effective Debye–Hückel interaction among themselves. The shielding parameter $\kappa$ reflects the effective range over which a single charge’s electric field is felt. Hence, the change in $\kappa$ also impacts the effective coupling strength of medium that has been quantified as $\Gamma ^{\star } = \Gamma \exp {(-\kappa )}$^41^ and later was improvised to $\Gamma ^{\star } = \Gamma (1+\kappa +\kappa ^2/2) \exp {(-\kappa )}$^52^. In both interpretations of $\Gamma ^{\star }$, the correlation gets weak with the increase in $\kappa$. We observe the increase in the growth rate of instability as $\kappa$ increases in molecular dynamics simulation of RTI. Figure 6a shows the evolution of RTI for three different values of screening parameter $\kappa = 0.1, 1$ and $\kappa = 2$. It is visible at early times, in the exponential growth regime, the growth rate $\gamma _e$ shows a slight enhancement with increasing $\kappa$. The differences in growth rate are small and well within the statistical error range. At later times, in the quadratic growth regime, the spike amplitude shows a significant increase in height ($H_S$) with the increasing $\kappa$. Table 1(left) lists out the calculated values $\gamma _e$ and $\gamma _q$ for three $\kappa$ values. As $\kappa$ varies $0.1 \rightarrow 2$, the growth rate significantly increase from $0.105 \rightarrow 0.127$ in the quadratic regime. We also observe the reduced and diffused mushroom-cloud formation at the tip of the spike with increasing shielding. This can be interpreted as the decrease in the effective coupling, leading to increased diffusivity.

### Effect of Atwood number $(A_t)$ on the growth of single-mode RTI

The Atwood number $A_t$ reflects the density contrast of heavy and light fluids. In the standard hydrodynamics, linear analysis suggests square-root dependence between the exponential regime growth rate and the Atwood number. Also, at higher values of $A_t$, spike penetration is significantly larger compared to the bubble rise due to the free fall. For SCPs, Fig. 6b shows the RTI growth for three values of $A_t = 0.3, 0.5$ and $A_t = 0.7$ from top to bottom rows. Corresponding growth rates in exponential, $\gamma _e$ and quadratic regime, $\gamma _q$ are shown in Table 1(right). We found the growth rate increasing with $A_t$ in both growth regimes. We also observe that the mushroom-cloud vortices are prominent for low $A_t$ values. This can result from more vertical resistance for the pair of less density-contrast fluids, causing the increased possibility of horizontal shear and, hence, prominent vortices.

**Table 1:** The exponential growth rate $\gamma _e$ and quadratic growth rate $\gamma _q$ of single-mode RTI (spike) at different shielding parameter $\kappa$ and Atwood number $A_t$.

Shielding parameter, $\kappa$	0.1	1.0	2.0	Atwood number, $A_t$	0.3	0.5	0.7
Exponential growth rate, $\gamma _e$	0.567	0.622	0.633	Exponential growth rate, $\gamma _e$	0.478	0.533	0.611
Quadratic growth rate, $\gamma _q$	0.105	0.124	0.127	Quadratic growth rate, $\gamma _q$	0.049	0.097	0.105

## Discussion

With the increase in coupling strength $\Gamma$, the plasma shows traits of solid. It remains in an intermediate state with coexisting features of fluid and solid before reaching a critical value $\Gamma _c$ at which the complete crystallization occurs. We have focused on this intermediate paradigm for a one-component shielded plasma, keeping the range of $\Gamma$ between 10 and 100. The main result is the suppression of the RTI growth rate with increasing coupling strength $\Gamma$ and may have many physical explanations. A possible physical mechanism is that the charged particles experience an increasing caging effect^53^. Now, any collective mode or dynamics has to invest more energy in taking particles out of their inertia under the confining potential. One may also expect the slow down against the increase in viscosity with coupling strength in the kinetic regime. We have validated our explanation based on this approach by calculating the hydrodynamic and kinetic viscosity domains’ growth rate and observed a consistent growth reduction from $\Gamma = 1$ to $\Gamma = 100$ values. To further support our explanation indirectly is to look into the effect of $\kappa$ on the growth rate. With increase in $\kappa$ (i.e., with decrease in effective coupling $\Gamma ^{\star }$), the growth rate increase. Our results regarding the suppression of RTI growth rate are also supported by the GHD model^38,54^. The visco-elastic GHD fluid model and molecular dynamics are the two useful approaches to analyze the collective dynamics in the intermediate coupling regime. For SCPs, the GHD model is useful in explaining the coexistence of longitudinal and transverse waves^55^. The description though qualitative, it has predicted convincing results related to the suppression of growth rate of instabilities like RTI^38,39^, KHI^56^, low-frequency modes in dusty plasmas^55,57^, coherent structures in strongly coupled plasmas^58^, and dynamic properties of SCPs^59^ in the linear dynamical regime. In nonlinear regime the phenomenological GHD model has also predicted the recurrences of KHI^20,56^, elastic-turbulence^60,61^ and cusp like structures^62^, which need a quantitative experimental and other simulation support. However, this model relies on MD simulations for transport coefficients for nonlinear dynamical studies. On the other hand, we attempt a realistic SCP model that includes all transport properties using a molecular dynamics simulation approach. MD also covers physics involvement through all possible scales from fluctuations to system size. The MD has been a realistic representative of SCPs^42–49^ for providing a better insight into collective processes when computation power is no more a restriction. Our MD simulations suggest that the strongly coupled plasma under intermediate states supports the fluid instability. This indicates that a quantitative fluid model, different from hydrodynamics and inline to GHD models, may be developed. Our results support the findings of the GHD model on the suppression of RTI in the linear regime. We further found that the suppression of growth rate with $\Gamma$ is also visible in the quadratic nonlinear dynamic regime. The MD results on suppression of RTI growth rate with $\Gamma$ is qualitatively supported by the GHD model predictions in the linear growth regime as in Das et al.^38^ and Avinash et al.^39^. We have not made any growth rate comparison in the nonlinear growth regime of dynamics.

**Figure 7:**
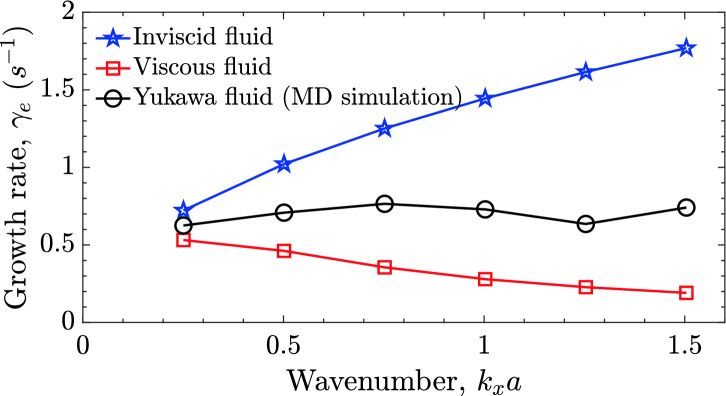
Comparison of dispersion relation of RTI for inviscid (blue stars), viscous (red squares) and Yukawa (black circles) fluids. Inviscid-hydrodynamic growth rate^3^ is $\gamma _e = \sqrt{g A_t k_x}$ and viscous-hydrodynamic growth rate (Eq. (8) in Ding et al.^23^) is $\gamma _e = \sqrt{\nu ^2 k_x^4 + g A_t k_x} - \nu k_x^2$, with shear viscosity $\nu$ taken from Donkó et al.^26^. Yukawa fluid growth rate is obtained from MD simulations for $\Gamma = 10$, $\kappa = 0.1$, and $A_t = 0.7$.

We study the growth of modes at different scales in the system through the dispersion relation. Once we established a way to calculate growth for single-mode, we extended our studies up to six modes in the system. We have given six perturbations each individually with $k_x = n k_0$, where $n=1, 2, \ldots , 6$ is the mode number (see Supplementary 2 for $k_x = 2$ and 3 modes). Each time, we have calculated the growth rate in the exponential and the quadratic regimes. Using the exponential growth rate data for different *k* values we draw the dispersion relation as shown in Fig. 7. We also plotted the dispersion relation of inviscid and viscous fluid for the comparison study. For the latter case, the value of viscosity is taken from MD simulation results in the literature^26^. As in our system configuration, the viscosity value is different from top to bottom region, and a mean value is used for fluid dispersion relation calculation. Figure 7 suggests that the hydrodynamic inviscid growth rate is higher compared to both the viscous as well as SCP. This is due to the lack of a damping mechanism in an inviscid fluid. However, the growth rate for the SCP is obtained to be higher than the pure viscous fluid. We do not have any definite understanding of the possible physical reason behind such difference. One possible reason can be thought of that some part of the viscous contribution is now being used towards the solid-like nature. We provide a probable qualitative explanation of why the RTI growth rate falls within the limits of inviscid and viscous fluids in the strong coupling. The explanation is based on the phenomenological GHD model. For the limit, $\tau _m \rightarrow 0$, the GHD model represents a viscous fluid. In the opposite limit of $\tau _m \rightarrow \infty$, the growth rate from Das et al.^38^ and Avinash et al.^39^ can be referred as $\gamma = \sqrt{g k_x A_t - \eta /\tau _m k_x^2}$. We see the growth rate will lead towards the inviscid hydrodynamic limit as $\tau _m \rightarrow \infty$. Thus, we may consider the growth rate values within viscous and inviscid limits for any strong coupling intermediate parameter regime. A similar result has earlier been reported for KHI using the GHD model^63^. For a given set of $\Gamma ,\kappa$, the growth dispersion relation for MD seems first to increase, reach an optimal value and then decrease. For $\Gamma = 10, \kappa = 0.1$, this optimal value reaches at $k_xa \sim 0.75$. The possible reason may be that the viscous effect is dominant over the growth of RTI compared to the low wavenumber regime for high wavenumbers. The GHD model does not quantitatively predict this optimal wavenumber. Though, qualitatively, the GHD model-based RTI growth rate $\gamma = \sqrt{g k_x A_t - \eta /\tau _m k_x^2}$ does predict an optimal growth rate as we increase the wavenumber. Brown et al.^50^ and Lyon et al.^51^ suggest that the plasma accesses the moderate coupling regime during the ICF process, a stage appears when density is large enough, the temperature has not raised enough^64–66^. Also, during the ICF process, the strong density gradient at the spherical capsule interface is prone to RTI when lasers squeeze the capsule from all directions. Suppression of RTI can be advantageous in such a parameter regime. This scenario is represented as strongly coupled and shielded ions in the present model. However, we must caution that our results include only electrostatic physics. Perturbations on specific modes at the interface in our studies can be visualized in line with the askew interface created by finite laser beam assembly. While present results guide the importance of strong coupling over the ICF process, full-scale modelling with spherical geometry, appropriate density difference, and acceleration caused by the laser assembly can provide a qualitative picture. Present studies can be an excellent test-bed to explore turbulent characteristics with RTI as a seed for nonlinear mixing. Direct particle-based modelling eliminates any grid-dependent scaling associated with fluid models. It is also a generalized approach that helps understand linear and nonlinear fluid processes lacking a quantitative fluid representation. Most rheological fluids fall under this category where fluid behaviour is far from Navier–Stokes governing dynamics. It would be interesting to know if Kolmogorov scales get altered for SCPs or how closely the turbulent scaling follows elastic turbulence features at low Reynolds number flows. Further, the kinetic simulations will help check the heating rate of the medium during the mixing as the energy eventually gets lost in the form of temperature. Finally, it will be worth comparing the computational cost incurred for kinetic and fluid models to visualize equivalent turbulence features. In the present work, we explicitly observe the development and progress of RTI in SCPs (or other representative Yukawa fluids) at different coupling strengths. A few open questions such as the effect of compressibility, roles of surface tension, and Reynolds number are under exploration and will be reported elsewhere. Compressibility could be a possible cause of sedimentation at high acceleration values. For now, we have significantly minimized it by reducing the acceleration due to gravity. We have tested the RTI for different gravity values to establish the elimination of sedimentation before choosing a value. A comprehensive study of sedimentation in Yukawa fluids is carried out by Charan et al.^22^ who reported asymmetry effect arising due to gravitation in the lighter fluid. We also studied the effect of dimensionality on presented growth rate values. We found that the effect of the coupling strength on the RTI growth in 3D simulations is also of a suppressing nature and is qualitatively similar to what we reported in 2D simulation results. To get an idea of how different can be the effect of strong coupling in a 3D system compared to the present 2D system, we have carried out a few 3D simulations keeping most of the features of plasma the same and only changing the 2D slab ($L_y = 10L_x$) to a 3D beam with ($L_y = 10L_x = 10L_z$). We changed the initial perturbation from a line sinusoidal perturbation to a similar form of sheet perturbation in 3D simulations. While the growth rates for the 2D and 3D cases are different, the impact of coupling strength is the same, i.e., the growth of instability decreases with the increase in the coupling strength (Figure  S1 in the Supplementary). Being molecular dynamics studies governed by electrostatic Coulomb potential and classical equation of motion, electromagnetic effects can’t be incorporated into our model. Such plasma modelling with the significant role of the self-consistently generated magnetic field and kinetic model is required and is done using Particle-In-Cell (PIC) simulations. However, PIC simulations are not suitable for explicitly looking into the strong coupling effects due to the small-angle collision approximation. Thus for motives of understanding strong coupling effects, molecular dynamics is an excellent simulation tool at the expense of heavy computation. Further, while our results represent all classical strongly coupled plasmas, the choice of the parameters is specific to dusty plasmas. For such systems, the time scales are very slow, and the velocity of heavy dust particles is slow enough that a self-generated magnetic field is insignificant for such a physical scenario. Also, to externally magnetize such a medium, an enormous magnetic field of about 4–10 Tesla is required, available only at a few facilities worldwide. The external magnetic field can be modelled in MD simulations by modifying the Velocity-Verlet algorithm. The same we will extend as future scope of our work.

## Methods

The classical molecular dynamics simulation is carried out using open-source Large-scale Atomic/Molecular Massively Parallel Simulator (LAMMPS) package^67^ for a system in which particles interact through repulsive Debye-Hückel/Yukawa potential as given by Eq. (1). The particle trajectories ${\textbf {r}}_i(t)$ are obtained by integrating the equation of motion $m \ddot{{\textbf {r}}}_i =\ - \nabla \sum \phi _{ij}$. The form of interaction potential and the charge on particle is same for all particles in the system.

**Figure 8:**
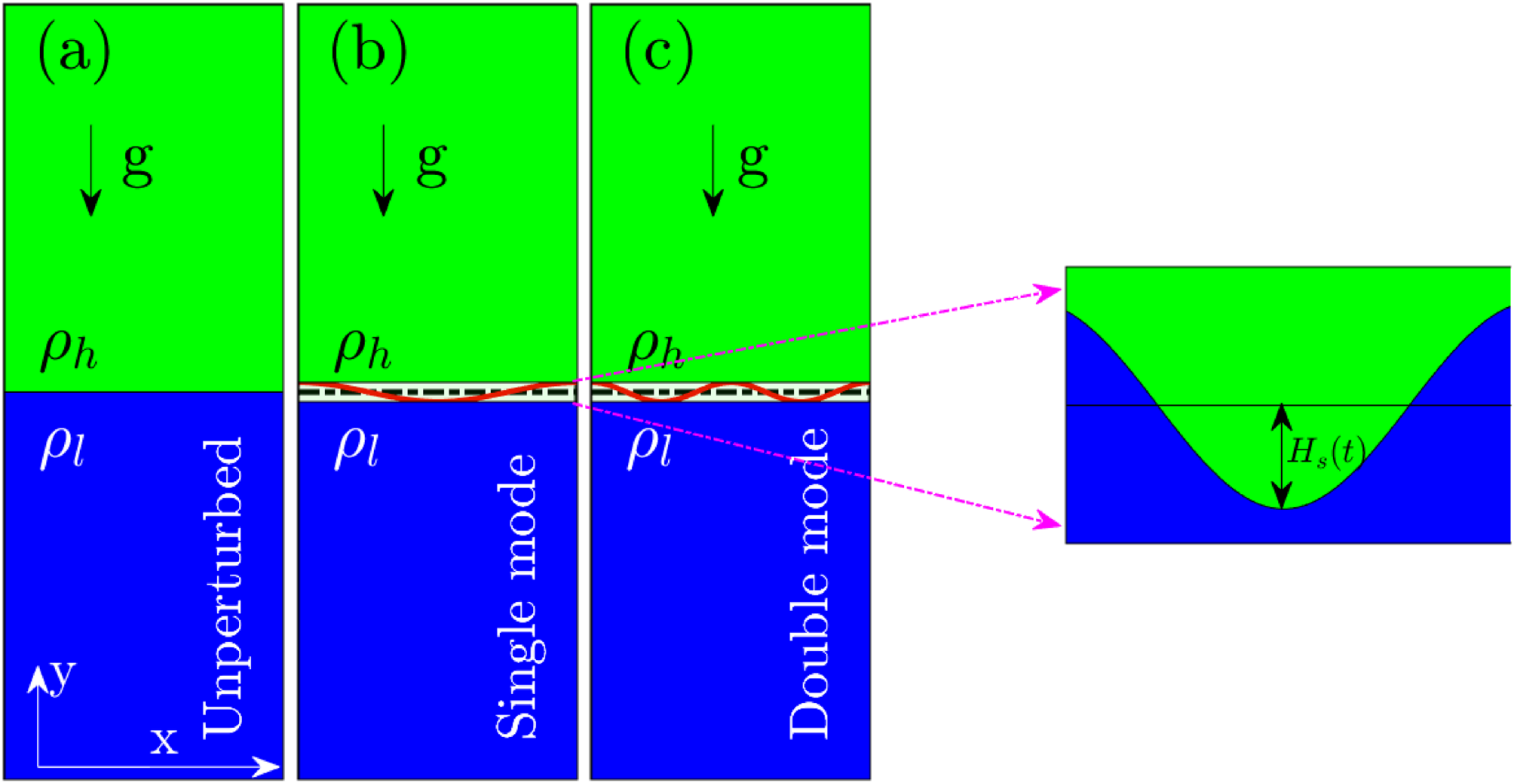
Schematic diagram used to study RTI in 2D $((0,L_x), (0,L_y))$ MD simulations using LAMMPS. (**a**) Initial system configuration with heavy fluid (density, $\rho _h$) on top of the light fluid (density, $\rho _l$). Single (**b**) and double (**c**) mode perturbation excitation at the interface between the heavy-light fluid (width = 100 a). The magnified view of the interface at the rightmost end shows the growth of the spike amplitude $H_s(t)$ of the single-mode initial sinusoidal perturbation.

**Table 2:** Simulation parameters for RTI.

Particle parameters	Rectangular configuration $(L_y = 10 L_x)$	Square configuration Fig. 1$(L_x = L_y)$
No. of particles, *N*	$2\times 10^5$	$5 \times 10^5$
Number density, *n*	$2.923\times 10^6$ m$^{-2}$	$2.923 \times 10^6$ m$^{-2}$
Charge on particle, *q*	$15\times 10^3$ e ; e = Electron charge $= - 1.6 \times 10^{-19}$C	$15\times 10^3$ e ; e = Electron charge $= - 1.6 \times 10^{-19}$C
Mass of lighter species, $m_l$	$6.9\times 10^{-13}$ kg	$6.9\times 10^{-13}$ kg
Acceleration due to gravity, *g*	$10^{-4}g_{Earth}$ ; $g_{Earth} = 9.81 m/s^2$	$10^{-4}g_{Earth}$ ; $g_{Earth} = 9.81 m/s^2$

A two-dimensional rectangular system in the x–y plane is configured, keeping periodic and reflecting boundaries in x and y directions, respectively. Throughout the paper, we have followed one particular system dimension (i.e., rectangular system except in Fig. 1) to keep the wave-number associated with single-mode perturbation identical. Thus, while comparing growth rates, we could focus on the effect on one parameter from $\Gamma$, $A_t$ and $\kappa$ at a time. Though the results are generalized and can be produced for any system dimension that can reflect collective dynamics. The system is divided into two regions top (high-density fluid $\rho _h$) and bottom (low-density fluid $\rho _l$), separated by a reflecting interface at the middle in the y-direction. The $\rho _h$ and $\rho _l$ can be expressed in terms of number density and mass through the relation $\rho _{s} = m_{s} n_{s}$ with $s = h,l$. In this work, the number density of both species is kept the same i.e., $n_h = n_l = n$ and the mass density has been changed through varying the mass of top and bottom fluids. The advantage of keeping number densities the same for heavy and light fluids is that the complete system remains at one $\Gamma$ value in the initial configuration. This configuration helps us understand the effect of coupling strength on instability growth. Thus, the Atwood number, $A_t$ depends on the difference of masses of both species. In simulations, we choose a value of $A_t$, fix the mass ($m_l$) of the light fluid and then calculate the mass of the heavy fluid using $m_h = m_l (1+A_t)/(1-A_t)$. $A_t$ ranges from 0 ($\rho _h = \rho _l$) to 1 ($\rho _h>> \rho _l$). The parameters used for the simulation are tabulated in Table 2. While the simulation parameters look unusual for hydrodynamic fluids and plasmas, they are a typical for any laboratory dusty plasma experiment. Our simulation parameter values are taken from the dusty plasma experiments^68,69^ where each heavy dust grain acquires large charge. Also, such dusty plasma experiments are often carried out in microgravity conditions and zero-gravity flight experiments where gravity values are close to what has been adopted in the present work. The system lengths and timescales have been normalized in terms of average inter-particle separation $a = (n \pi )^{-1/2}$ and plasma period $\omega _{pl}^{-1}$ of light species. While the simulation results apply to any liquid with Yukawa form of inter-particle interaction potential, the normalization of timescales is motivated by its plasma representation where $\omega _{pl} = \sqrt{(n_l q^2/\epsilon _0 m_l 2a)}$ is the characteristic plasma frequency of the light fluid.

Figure 8a shows the initial two-fluid system configuration with step mass density profile at the interface in the y-direction. The gravity is in the negative y-direction. Particles of heavy and light masses are created randomly and homogeneously in top and bottom regions, respectively. Both density regions (i.e., top and bottom in Fig. 8) have been independently equilibrated using Nosé–Hoover^70,71^ thermostat for 400 $\omega _{pl}^{-1}$, enough time for both regions to attain the required temperature hence the coupling strength. Further, we detached the thermostats and let the system evolve under an NVE ensemble condition for the next 400 $\omega _{pl}^{-1}$. During this phase, we observed no heating, a reflection of a naturally equilibrated system. At this stage, the system is ready for RTI studies. Under the NVE conditions, we remove the interface between heavy and light fluids under gravity and let the instability evolve. A maximally growing mode will appear unstable from natural perturbations. To study the single mode or double mode instability growth specifically, we apply weak artificial perturbation as shown in Fig. 8b,c.

## Supplementary Information

Supplementary Information.
