# Supplementary material for: Rayleigh–Taylor instability in strongly coupled plasma
Source: Sci Rep. 2022 Jul 7;12:11557. doi: 10.1038/s41598-022-15725-2 (PMC9262965; doi:10.1038/s41598-022-15725-2)
Supplement: Supplementary file 1 — Supplementary Information. [file 41598_2022_15725_MOESM1_ESM.pdf]

Rayleigh-Taylor Instability in Strongly Coupled Plasma

Rauoof Wani[1,+], Ajaz Mir[1,+], Farida Batool[1], and Sanat Tiwari[1,*]

[1]Indian Institute of Technology Jammu, Department of Physics, Jammu, 181221, India

[*]sanat.tiwari@iitjammu.ac.in

[+]Equal contribution

SUPPLEMENTARY 1: Growth rate of three-dimensional single-mode RTI in SCP

Here we demonstrate that the strong coupling has similar growth rate reduction attributes in 2D and 3D systems. Figure S1 shows suppression of the exponential γ_e as well as the quadratic γ_q growth rate in the 3D system with increasing Coulomb coupling strength Γ. To prepare a 3D system, we have taken a slab assembly with $L_y = 10L_x = 10L_z$, keeping the height of the slab the same as for 2D systems studied in detail. The perturbation has also been given a sheet sinusoidal form for single-mode perturbation. The density of the 3D system is chosen such that the average inter-particle separation remains the same for 2D and 3D system assembly. This also helps us keep the coupling strength Γ same to make a comparison convenient.

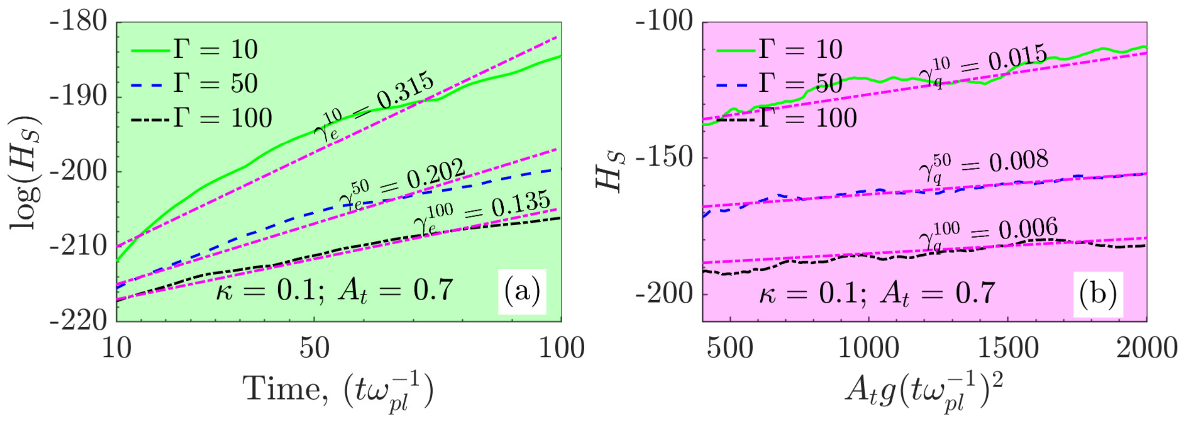

Figure S1 . Exponential and quadratic growth rate of the single-mode RTI in 3D simulation at $\Gamma = 10$, 50 and $\Gamma = 100$. Both the exponential γ_e as well as the quadratic γ_q growth rates decrease with increasing coupling strength Γ.

SUPPLEMENTARY 2: Double and triple-mode RTI excitation in SCP

The dispersion relation shown in Fig. 7 includes the growth of 2D RTI for a given $\Gamma = 10$ and different values of wavenumber, k_x, including single, double, triple, up to six modes accommodated in the x-direction. The single-mode instability growth has been clearly demonstrated in Figs 2, 3, 4 and 6. Here we provide the explicit growth of RTI for double and triple mode cases from where the growth rate values have been deduced for the dispersion relation. The growth of particular single, double and triple modes through perturbation is visible from Figs. 2 and S2.

SUPPLEMENTARY 3: Governing equation of motion for the particles

The particle trajectory $\mathbf{r}_i(t)$ of each particle is obtained by integrating the equation of motion

$$m\ddot{\mathbf{r}}_i = -\nabla \sum \phi_{ij} \tag{1}$$

The potential for the Debye-Hückel governed charged fluid is given by

$$\phi_{ij} = \frac{1}{4\pi\varepsilon} \frac{q_{ij}^2}{r_{ij}} \exp\left(-\frac{r_{ij}}{\lambda_D}\right) \tag{2}$$

and for the Yukawa fluids the potential form is given by

$$\phi_{ij} = \frac{A}{r_{ij}} \exp\left(-\frac{r_{ij}}{\lambda_D}\right) \tag{3}$$

Both forms of potential are equivalent with $A = q_{ij}^2/4\pi\varepsilon_0$.

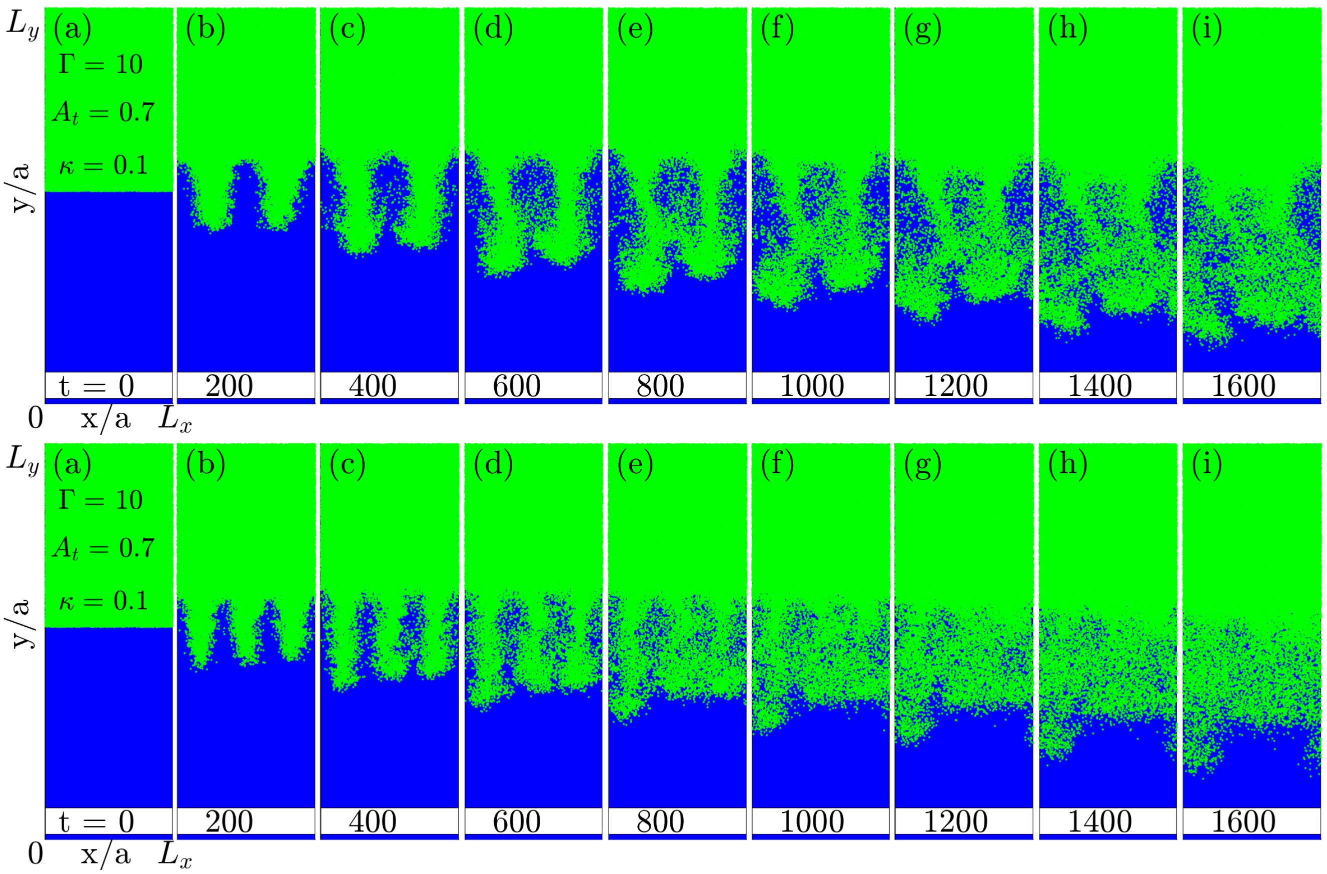

Figure S2 . Time evolution of double and triple-mode of RTI from $t = 0\ \omega_{pl}^{-1}$ to $t = 1600\ \omega_{pl}^{-1}$. Top panel: Double-mode spikes of heavier fluid penetrate the lighter fluid due to gravity while as light fluid bubbles rise up due to buoyancy. Bottom panel: Triple-mode penetration of spikes and rise of bubbles in the two fluid configuration. The double and triple-modes are generated due to sinusoidal perturbation having primarily two and three bumps and depressions.
